# An Historical Sketch of the State of Medicine in the American Colonies

**Published:** 1850-06

**Authors:** 


*An Historical Sketch of the State of Medicine in the American Colonies, from their first Settlement to the Period of the Revolution.* By John B. Beck, M. D., Professor of Materia Medica and Medical Jurisprudence in the College of Physicians and Surgeons, in the city of New York, &c. Second edition. 1850.

We cordially thank Dr. Beck, for this interesting brochure, the title of which heads these romarks. The author has redeemed much valuable matter from oblivion, and presents it to the profession in a fascinating form. The pamphlet contains a record of the pioneers of the medical profession, on this continent, and of many noble spirits and burning lights in their ranks. As a contribution towards a history of the North American medical profession, we hail this pamphlet with sincere satisfaction. The sketches of Drs. Morgan, Lloyd, Wm. Shippen, Mitchell, of Virginia; Lining, Douglass, of Boston; Ogden, Thomas, of Virginia; Manson, Bayley, Chalmers, Gardue, Clayton, of Virginia; Tennent, a relation of Dr. Richard Meade, of London; Cadwallader, Bond, Colden, Bard, Middleton, Jones, of New York; are full of interest. We acknowledge ourself indebted to Dr. Beck, for some delighted reading hours.

